# Effects and Mechanisms of TikTok Use on Self-Rated Health of Older Adults in China During the COVID-19 Pandemic: A Mediation Analysis

**DOI:** 10.3390/healthcare12222209

**Published:** 2024-11-06

**Authors:** Yunfeng Luo, Han Yu, Yalin Kuang

**Affiliations:** School of Public Administration, University of Electronic Science and Technology of China, Chengdu 611731, China; yunfengluo@uestc.edu.cn (Y.L.); yuhannuts@163.com (H.Y.)

**Keywords:** TikTok use, self-rated health, older adults, COVID-19, exercise frequency, protein intake

## Abstract

Background/Objectives: During the COVID-19 pandemic, TikTok emerged as a significant app for entertainment and information acquisition for the general public, gradually extending its reach to the older population. Digital technology, exemplified by TikTok, has become an indispensable part of older adults’ lives. However, while prior studies have extensively focused on the impact of internet use on older adults’ health, research on the impact of TikTok during the COVID-19 pandemic remains insufficient. Methods: Utilizing data from the China Family Panel Studies (CFPS) 2020, this study explored the impact of TikTok use on older adults’ self-rated health and its underlying mechanisms through multiple regression and mediation analysis. Results: The study found that (1) TikTok use had a significant positive effect on the self-rated health of older adults; (2) while TikTok use increased the exercise frequency of older adults, exercise frequency did not affect their self-rated health; and (3) TikTok use boosted older adults’ protein intake, contributing substantially to their self-rated health, accounting for 38.7% of the total effect. Conclusions: It is advisable to actively promote the popularity of short video apps such as TikTok among older adults, as they serve as an effective tool for disseminating health information and encouraging healthier lifestyles and behaviors.

## 1. Introduction

As projected by the World Health Organization (WHO), approximately one-sixth of the global population is expected to be aged 60 years or older by 2030, presenting a significant challenge for countries worldwide [1]. In the context of an aging population, the health of older adults is particularly critical [2]. The COVID-19 pandemic, which emerged in 2019, resulted in an unprecedented health crisis [3]. Although the virus affected individuals across all age groups, evidence indicated that the elderly population faced a heightened risk of mortality [4]. Mortality rates among older adults in China, Western Europe, the United Kingdom, Canada, and the United States were 8 to 62 times higher than those of adults under 55 years, with the highest rates observed among individuals aged 65 and above [5].

Simultaneously, there has been a significant rise in the popularity of short video applications, particularly TikTok, which is widely used in 155 countries and regions, boasting over 800 million active users and 2 billion downloads globally [6]. During the COVID-19 pandemic, TikTok’s popularity surged, making it the most downloaded app of 2020 [7,8]. In December 2020, it was reported that over two-thirds of consumers in the United States spent at least 30 min and up to three hours per day viewing short videos [9]. Additionally, the user base of short video apps is gradually expanding to include older adults [7]. TikTok reported that a substantial proportion of the 152 million new users in March 2020 were older adults [10].

Digital technology has become a vital aspect of older adults’ lives [11], potentially serving as a significant factor influencing their health. On one hand, social media platforms, such as TikTok, play a crucial role in facilitating access to health information and enhancing health literacy among older users [12]. The use of social media platforms positively impacts public awareness of health measures and preventive behaviors [13,14,15,16,17]. Recent studies have shown that individuals who engage with social media platforms are more likely to adhere to hygiene protocols in response to the COVID-19 pandemic [18] and more inclined to adopt protective measures, such as practicing social distancing [19], compared to those who do not use such platforms. On the other hand, as a quintessential hedonistic social media platform, TikTok is designed to retain users for extended periods [20], which may lead older users to develop an addiction to the online world [21], posing potential risks to their health [22]. Addiction to social media can result in physical issues such as eating disorders [23] and sleep disturbances [24] among older adults, as well as mental health challenges including anxiety and depression [25].

Therefore, this study utilized data from the CFPS 2020 to explore the effects of TikTok on the self-rated health of older adults during the COVID-19 pandemic and the mechanisms underlying its impact.

## 2. Literature Review

### 2.1. TikTok Use and Health of Older Adults

**Research question 1**: What is the potential relationship between TikTok use and the health of older adults?

When it comes to the relationship between Internet use and the health of older adults (in accordance with the WHO definition, the term “older adults” is used to describe individuals aged 60 and above in this study), one perspective is that Internet use positively impacts the health of older adults, supported by network gain effect theory, which suggests that Internet use can help older adults expand their existing social networks, establish additional social interactions, and acquire new human capital, thereby benefiting their physical and mental health [26,27]. Conversely, another view argues that Internet use may be unrelated to older adults’ health and could even be harmful. This perspective is based on presence substitution effect theory, which posits that Internet use displaces offline social interactions and physical activity time [28], potentially narrowing social networks, fostering detrimental habits, and negatively affecting health [29].

Beyond methodological and data differences, a significant factor contributing to these varying findings is the heterogeneity in the ways the Internet can be used. In China, 88.3% of Internet use is attributed to the viewing of short videos [30]. Consequently, the impact of TikTok use on health has become a hot topic in scholarly fields. For instance, Wang and Scherr [31] have noted the potential negative impact of TikTok on sleep health among Chinese users, while Basch et al. [32] have highlighted TikTok’s potential to improve population health due to its unique information dissemination and interactive features. Liang and Pang [33] have demonstrated that short videos can enhance health awareness and encourage vibrant lifestyles. Additionally, short videos have been shown to motivate older women to engage in physical activity. Ramsden et al. [34] have indicated that TikTok can help alleviate stress, reduce negative emotions, and promote positive health outcomes. However, it is crucial to recognize that such platforms may also lead to upward comparisons, potentially resulting in negative emotions like low self-esteem, which could adversely affect health. Samuel et al. [35] have also emphasized TikTok’s effectiveness as a health promotion tool for influencing weight loss. Given the literature and theories, this study proposed a series of competing hypotheses:

**Hypothesis 1-0.**  *The use of TikTok negatively influences or does not influence older adults’ health*.

**Hypothesis 1-1.**  *The use of TikTok positively influences older adults’ health*.

### 2.2. Impact Mechanisms

**Research question 2**: How does TikTok use potentially affect the health of older adults?

Short video apps are effective tools for individuals to obtain health information [36]. During the COVID-19 pandemic, as of July 2020, TikTok videos related to the pandemic had garnered 93.1 billion views [37]. Older adults access health information through short videos, which positively influence their health-related behaviors and habits [38]. The health information accessed by older adults via TikTok predominantly pertains to exercise and diet [39].

Regarding exercise, a study by Davies et al. [40] demonstrated that social media platforms like TikTok significantly influence users’ exercise behaviors. Notably, during the COVID-19 pandemic, there was a marked increase in public awareness of the importance of boosting immunity, accompanied by a surge in the demand for physical activity. TikTok played a significant role in promoting physical activity during self-quarantine, with online training sessions by fitness experts and influencers providing opportunities for individuals to engage in exercise [41]. When older adults are unable to participate in outdoor activities such as walking or square dancing, viewing TikTok videos encourages them to maintain their exercise routines [42]. Physical activity has been shown to be an effective way of maintaining a healthy weight, improving cardiovascular health, and alleviating stress and anxiety [43].

Conversely, addiction to TikTok may lead to feelings of loneliness [44], depression [45], and prolonged periods of sedentary behavior [46], ultimately resulting in an unhealthy lifestyle. Furthermore, TikTok use has the potential to create a time-crowding effect, reducing the time available for exercise and sleep, which may cause older adults to be less physically active. For instance, Wu et al. [9] found that increased exposure to short videos was significantly associated with a decrease in the number of steps taken and the minutes spent engaged in physical activity. And physical inactivity is a significant contributing factor to the prevalence of various chronic diseases [47], which can adversely affect the health of older adults. Accordingly, the following hypotheses were formulated:

**Hypothesis 2-0.**  *TikTok use has a negative or no effect on older adults’ health by influencing their exercise frequency.*

**Hypothesis 2-1.**  *TikTok use has a positive effect on older adults’ health by influencing their exercise frequency.*

Regarding diet, the primary contributors to the rising incidence of obesity and chronic diseases are unhealthy eating habits, such as excessive energy intake, a high consumption of saturated fats and trans-fatty acids, a low intake of fruits and vegetables, and excessive salt consumption [48]. Older adults can improve their eating habits by accessing a diverse range of healthy eating advice through short video apps. Xue et al. [49] found that Internet use led to increased energy, protein, and fat intake, as well as improved health among individuals in rural areas. Additionally, TikTok live-streaming can help alleviate consumers’ budgetary constraints by reducing the price of goods and transaction costs, thereby increasing spending on high-quality foods [50]. Drivas et al. [51] observed that viewing food-related videos on TikTok led to noticeable changes in individuals’ dietary habits. Therefore, the following hypotheses were formulated:

**Hypothesis 3-0.**  *TikTok use has a negative or no effect on older adults’ health by optimizing their eating habits.*

**Hypothesis 3-1.**  *TikTok use has a positive effect on older adults’ health by optimizing their eating habits.*

## 3. Research Design

### 3.1. Data Sources

The CFPS is a comprehensive nationwide multidisciplinary tracking survey project that has been organized by the Institute of Social Science Survey at Peking University since 2010. The project spans 25 provinces across China, collecting data from three dimensions: individual, family, and community. It employs various data collection methods, including long, short, pick-up, and telephone interviews, to capture changes in China’s social, economic, demographic, health, and educational landscape.

The data used in this study were sourced from CFPS 2020, the most recent publicly available dataset, following the COVID-19 pandemic in 2019. The study focused on a sample of older adults aged 60 and above, resulting in 6784 valid samples.

### 3.2. Variables

Dependent variable. The study adopted the methodology of Du and Wang, using self-rated health as a measure of health status [52]. The questionnaire asked, “How do you think your health is?”, with responses given on a five-point Likert scale from “very unhealthy” to “very healthy”, scored from 1 to 5. As shown in Table 1, the average self-rated health of older adults in China is 2.492, suggesting that a significant portion of Chinese older adults reported suboptimal health.

Independent variable. The independent variable was TikTok use, assessed by the question, “Have you watched any short video apps such as TikTok in the past week?”.

Mediating variables. This study considered two mediating variables based on previous hypotheses. First, the exercise frequency was measured with the question, “Excluding cycling and walking for work, how often do you participate in sports and fitness activities (sports and fitness activities refer to indoor and outdoor physical activities for the purpose of physical fitness and the enjoyment of body and mind) in the past year?”. This was scored on an 8-point scale from 0 to 7, with higher scores indicating more frequent exercise. The mean score was 1.954, indicating that most Chinese older adults engage in low levels of exercise. Second, a healthy diet includes adequate protein intake, crucial for older adults’ physical functioning and overall health [53,54]. Protein intake was characterized by the question, “Did you eat meat in the past week?”, with a “yes” response coded as 1 and “no” as 0. In the 2020 survey, approximately 78.9% of elderly Chinese people consumed protein, reflecting their dietary habits.

Control variables. The study controlled for variables potentially affecting older adults’ health, identified at the individual, familial, and geographic levels. Individual-level variables included age, gender, marriage, subjective social status, subjective economic status, BMI, and sleep duration, with quadratic terms of BMI and sleep duration added to account for possible nonlinear effects. Familial-level variables comprised the number of family members and the ratio of family medical expenditure, indicating family support and the financial burden of healthcare. Geographic-level variables included the urban–rural and geographic region, reflecting the influence of the household registration system and regional development disparities. According to Table 1 and Table 2, the mean age of older adults in the study was 70 years, with a nearly equal gender distribution and a higher proportion of married individuals (82.9%). The mean subjective social and economic status scores were 3.483 and 3.191 (out of 5), respectively, indicating a relatively high social and economic status. The mean BMI was 23.2, within the healthy range. The mean family medical expenditure ratio was 13.9%, while 47.1% of older adults resided in urban areas. The average sleep duration per day was 7.3 h.

### 3.3. Empirical Model

In this study, a multiple regression model was constructed to test the effect of TikTok on the self-rated health of older adults. The multiple regression equation is presented in Equation (1).

(1) $$H_{i}=\beta_{0}+\beta_{1}T_{i}+\beta_{2}X_{i}+\varepsilon_{i}$$

where $H_{i}$ represents the self-rated health of older adults, $T_{i}$ indicates whether or not they use TikTok, $X_{i}$ is the control variables that encompass individual, familial and geographic-level variables, $\varepsilon_{i}$ is a random error term, and $\beta_{1}$ and $\beta_{2}$ are estimated coefficients.

To gain a deeper understanding of the underlying mechanisms, this study utilized stepwise regression [55] to assess potential mediating effects. First, it estimates whether the independent variable significantly affects the dependent variable. Second, it evaluates whether the independent variable significantly influences the mediating variables. Finally, it estimates the regression model by including both the independent variable and the mediating variables. The model specifications are illustrated in Equations (2)–(4).

(2) $$H_{i}=\alpha_{0}+\alpha_{1}T_{i}+\alpha_{2}X_{i}+\varepsilon_{i}$$

(3) $$M_{i}=\gamma_{0}+\gamma_{1}T_{i}+\gamma_{2}X_{i}+\varepsilon_{i}$$

(4) $$H_{i}=\delta_{0}+\delta_{1}T_{i}+\delta_{2}M_{i}+\delta_{3}X_{i}+\varepsilon_{i}$$

where $M_{i}$ represents mediating variables, including exercise frequency and the protein intake of older adults, and $\delta_{1}$ and $\delta_{2}$ are the coefficients of the effect of TikTok use and mediating variables on self-rated health, respectively, after controlling for various factors. If $\alpha_{1}$, $\gamma_{1}$ and $\delta_{2}$ are significant, the mediating mechanism is considered valid. In this case, if the effect of $\delta_{1}$ is not statistically significant, it can be concluded that there is a complete mediation effect. If the effect of $\delta_{1}$ is statistically significant, and $\delta_{1}\;$<$\;\alpha_{1}$, this indicate that there is a partial mediation effect.

## 4. Results

### 4.1. Multiple Regression

Table 3 presents the results of the multiple regression analysis.

The results indicated that older adults who use TikTok have self-rated health scores 0.125 points higher than those who do not use TikTok, with this difference being significant at the 5% level. This finding suggests that TikTok use significantly improves self-rated health among older adults in China.

Additionally, age was negatively correlated with self-rated health (*β* = −0.012, *p* < 0.01), aligning with the established pattern of human growth observed in previous studies [56]. Men reported higher self-rated health compared to women (*β* = 0.215, *p* < 0.01), consistent with the “health–survival” paradox proposed by Archer (2018), where women live longer but have worse health than men [57]. The study also found that an increase in economic status was associated with better self-rated health among the elderly (*β* = 0.180, *p* < 0.01), likely due to improved access to quality healthcare resources and the financial ability to pursue a healthier lifestyle. The relationship between sleep duration and self-rated health followed an inverted U-shaped pattern (*β* = 0.284, β of quadratic term = −0.018, *p* < 0.01), with the optimal sleep duration being approximately 7.9 h. Prior research supports that adequate sleep duration is a key determinant of good self-rated health [58,59]. Furthermore, the number of family members was positively associated with self-rated health (*β* = 0.019, *p* < 0.05), potentially due to an enhanced family support system providing emotional comfort and practical assistance to the elderly [60]. The ratio of family medical expenditure was inversely correlated with self-rated health (*β* = −0.424, *p* < 0.01), likely due to health shocks leading to increased medical care demands and higher family expenditure. Urban elderly individuals reported significantly better self-rated health compared to their rural counterparts (*β* = 0.095, *p* < 0.05), and elderly individuals in the western region had significantly lower self-rated health compared to those in the eastern region (*β* = −0.149, *p* < 0.01), which may be related to the unequal distribution of healthcare resources [61,62].

### 4.2. Mediating Effects Test

The results presented in Table 4 demonstrate that TikTok use was associated with an increase in the frequency of exercise among older adults (*β* = 1.037, *p* < 0.01). However, this increase in exercise frequency did not significantly impact the self-rated health (*β* = 0.009, *p* > 0.1). Additionally, TikTok use was linked to a higher probability of protein intake among older adults (*β* = 0.628, *p* < 0.01), which exhibited a significant positive effect on their self-rated health (*β* = 0.120, *p* < 0.05). This finding suggests that protein intake partially mediates the relationship between TikTok use and self-rated health among older adults.

**Figure 1:**
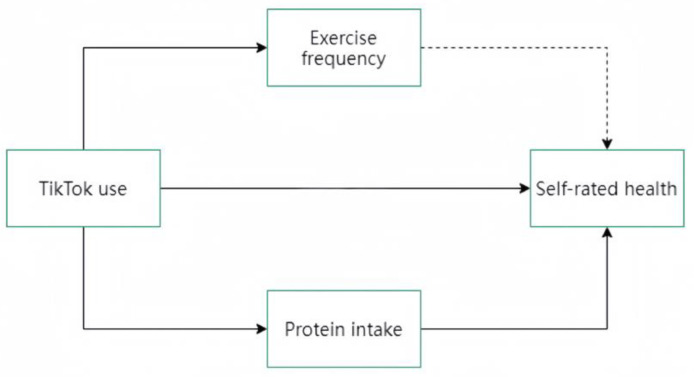
Diagram of parallel mediation.

Figure 1 shows the framework of parallel mediating effects, with dashed lines indicating the absence of a significant effect.

The parallel mediating effects estimated from the generalized structural equation model are presented in Table 5. The results of Table 5 demonstrated that the 95% confidence intervals for the direct effect, indirect effect 2 (protein intake) and the total effect did not pass through the 0 point and were statistically significant. The mediating effect of protein intake was approximately 38.7% of the total effect, calculated as (0.058/0.150 × 100%).

As a consequence, Hypothesis 1-0, 2-1 and 3-0 were not corroborated by the findings, while Hypothesis 1-1, 2-0 and 3-1 were supported by the findings.

### 4.3. Robustness Test

Two distinct methodologies for the robustness test were employed in this study. First, recognizing that self-rated health may be influenced by subjective factors, such as positive attitude, which can lead to an overestimation of one’s health status, this study incorporated subjective well-being as a proxy variable for these factors.

Second, the dependent variable was replaced by the ability to perform activities of daily living (ADL), assessed based on whether respondents were able to independently engage in activities such as going outdoors, eating, cooking, taking public transport, shopping, bathing, and doing laundry. Each of these activities was assigned a score of one for a “yes” response, resulting in a total score ranging from 0 to 7. The sample mean was 6.452, with a standard deviation of 1.289.

The findings in Table 6 indicate that even after controlling for subjective factors, TikTok use continued to exert a significant positive effect on the self-rated health of older adults (*β* = 0.090, *p* < 0.1). And the replacement of the dependent variable had no impact on the results (*β* = 0.110, *p* < 0.05), thereby reinforcing the robustness of our conclusion.

### 4.4. Endogenous Test

It is acknowledged that the sample may be subject to a self-selection problem, whereby the group of older adults who use TikTok may possess certain characteristics that impact their self-rated health, leading to a misestimation of the impact of TikTok use. For this reason, the impact of TikTok use was estimated anew using a treatment effect model [63]. This was achieved by two main steps. In the initial step, the sample-selecting model was estimated using the probit model, from which the inverse Mills ratio (IMR) was calculated. In the subsequent step, the IMR was incorporated into the multiple regression model to estimate the coefficients. It is important to note that the control variables in the initial step of the probit model did not include BMI, sleep duration or familial-level variables. However, all control variables were incorporated into the subsequent step.

The results of Table 7 demonstrated that the coefficient of IMR was statistically significant at the 5% level (*β* = −0.502, *p* < 0.05), indicating the existence of sample self-selection. When the sample self-selection bias was circumvented, the effect of TikTok use on self-rated health still remained positive (*β* = 1.033, *p* < 0.05).

## 5. Discussion

In summary, (1) TikTok use had a significant positive impact on the self-rated health of older adults; (2) while TikTok use increased the exercise frequency of older adults, exercise frequency did not significantly affect their self-rated health; and (3) TikTok use enhanced older adults’ protein intake, which had a substantial positive impact on their self-rated health.

The findings indicated the significant positive effect of TikTok use on the health of older adults. This finding not only corroborates and deepens the established research findings on the impact of the Internet on the health of older adults [64,65], but also validates and expands the network gain effect theory. In recent years, short video apps have become the primary means of internet access for older adults, with a notable impact on their daily lives and social relationships [66]. In comparison to media platforms with text and images, short videos apps are more interesting and livelier. This format of presentation is more readily accepted by the elderly [67]. Indeed, many elderly users have turned to short videos [68] as a means of coping with the challenges posed by the pandemic. This study provides evidence for the potential use of TikTok as an effective tool for promoting health management among older adults during a health crisis.

The use of TikTok was observed to enhance the frequency of exercise among older adults. In China, older adults rarely engage in professional and comprehensive fitness programs, due to financial constraints or physical limitations. However, the advent of short video platforms has provided them with a cost-free avenue to pursue fitness [69]. The fitness influencers on TikTok come from a diverse range of backgrounds, including singers, actors, police officers, full-time housewives and security guards, etc. Despite not being professionals, they possess considerable experience in the field of fitness, and their basic qualifications are verified by TikTok, thereby ensuring their credibility [70]. And their fitness programs are typically designed for the general public, offering a range of intensity levels, which are also suitable for older adults. Furthermore, although not all older adults adopt fitness programs, relevant data indicate that fitness influences have a surprising and growing influence. For example, Liu Geng Hong, a Chinese star, began livestreaming exercises at home every day on TikTok during the COVID-19 pandemic, and within ten days, he had nearly 30 million fans [71]. In the context of the Internet age, mass fitness should consider not only the intrinsic value of health but also the value of mass participation.

However, the observed increase in exercise frequency did not yield a statistically significant impact on the health of older adults. There are two possible reasons for this discrepancy. First, the increase in exercise frequency may not reflect changes in exercise intensity. An increase in exercise frequency, without a corresponding increase in intensity, often results in limited health benefits. The WHO recommends that older adults engage in at least 150 min of moderate-intensity physical activity or at least 75 min of vigorous-intensity physical activity per week. However, few of them meet these guidelines [9], with the majority engaging in low-intensity physical activity in their daily lives [72]. Therefore, although TikTok has played a positive role in promoting older adults’ participation in physical activity, its effects may be difficult to fully realize if it fails to guide them to change the intensity of their exercise. In addition, a comprehensive health assessment is necessary prior to modifying any fitness program. Second, the COVID-19 pandemic has resulted in a significant shift towards indoor exercise among the elderly population, which may potentially restrict the diversity and efficacy of their exercise routines due to spatial limitations and inadequate equipment. Furthermore, mental health issues, such as anxiety and depression, which are associated with long-term self-quarantine among the elderly [73], may serve to negate the positive effects of physical exercise.

The use of TikTok increased protein intake among older adults, which had a significant positive impact on their health, accounting for 38.7% of the total impact. This finding suggests that protein intake partially mediates the relationship between TikTok use and the self-rated health of older adults. This underscores the importance of enhancing dietary habits for the continued well-being of older individuals during public health emergencies. Furthermore, although the investigation was limited to protein intake, it is unlikely that older adults will exclusively purchase protein-rich foods on TikTok, given the necessity of consuming a balanced diet. The results actually emphasized the significant contribution of TikTok to fostering healthy dietary practices. The prevalence of malnutrition among older adults represents a significant health risk [74], particularly in the context of the COVID-19 pandemic, which exacerbates the vulnerability of older adults to viral attacks. The prevalence of malnutrition was reported to be exceedingly high among older adults admitted with a diagnosis of COVID-19 [75]. The use of TikTok has the potential to expand older adults’ access to information on healthy diets, increase their awareness of the importance of health investments, provide them with more cost-effective and high-quality food, and help them develop healthy eating habits [76].

## 6. Policy Implications

The conclusions present significant policy implications. First, promoting the widespread and rational use of TikTok among older adults is essential. This involves enhancing the digital literacy of older adults through community centers [77], enabling them to acquire the skills necessary to navigate TikTok effectively and adapt to the digital age. Furthermore, it is also important for TikTok to improve app design by integrating larger fonts and voice commands, which can enhance accessibility and experience for older users [78]. Second, TikTok can be utilized to promote healthier lifestyles among older adults by establishing official health channels [79] that emphasize nutritious eating and protein intake. Providing online fitness classes specifically tailored to older adults and encouraging health professionals to share personalized advice can further elevate their health awareness [80]. Third, it is vital to strengthen community support and resource integration. Establishing health service platforms [81] on TikTok to connect older adults with health advisors and nutritionists, partnering with catering services for nutritious meal delivery and creating online health communities for shared experiences and mutual support.

## 7. Limitations

It is important to acknowledge that this study has limitations. First, this study only considered TikTok use as a criterion for evaluation; however, the frequency and duration of TikTok use may have varying impacts on older adults’ health, representing an important avenue for further investigation. Second, this study focused solely on the impact of exercise and diet in relation to TikTok use and the health of older adults. Future research would benefit from incorporating additional perspectives. Third, CFPS is a large-scale social survey rather than a specialized nutrition and health survey, and due to the principle of confidentiality, authors are unable to contact the respondents. Therefore, it is challenging to obtain detailed information about protein intake and exercise. Fourth, the dataset was limited to the year 2020, which restricts the generalizability of the conclusions. To enhance the reliability of the findings, future studies should consider using data from multiple time periods to understand the long-term effects of TikTok on older adults’ health.

## 8. Conclusions

The study utilized CFPS 2020 data to explore the impact of TikTok use on the self-rated health of older adults. The primary findings were as follows: first, TikTok use had a significant positive impact on the self-rated health of older adults; second, while TikTok use increased the exercise frequency of older adults, exercise frequency did not significantly affect their self-rated health; and third, TikTok use enhanced older adults’ protein intake, which had a substantial positive impact on their self-rated health, accounting for 38.7% of the total effect. It is advisable to actively promote the popularity of short video apps such as TikTok among older adults, as they serve as an effective tool for disseminating health information and encouraging healthier lifestyles and behaviors.

## Figures and Tables

**Table 1:** Variables and descriptive statistics.

Variables	Definitions	Mean	S.D.
Independent variable			
TikTok use	Use = 1, not use = 0		
Mediating variables			
Exercise frequency	How often do you participate in sports and fitness activities: 0 (never) to 7 (very often)	1.954	2.777
Protein intake	Did you eat meat in the past week: Yes = 1, no = 0		
Dependent variables			
Self-rated health	You think your health is: 1 (very unhealthy) to 5 (very healthy)	2.492	1.252
Control variables			
Age	Measured in years (above 60)	70.079	7.350
Gender	Male = 1, female = 0		
Marriage	Married = 1, unmarried = 0		
Subjective social status	Measured on a scale of 1 (low) to 5 (high)	3.483	1.110
Subjective economic status	Measured on a scale of 1 (low) to 5 (high)	3.191	1.160
Body Mass Index (BMI)	Calculated based on height and weight	23.202	3.539
Sleep duration a day	Measured in hours	7.248	1.633
Number of family members	Measured in persons	3.793	2.196
Ratio of family medical expenditure	Family medical expenditure/Total family expenditure	0.139	0.340
Urban	Urban = 1, rural = 0		
Geographic region	Eastern region = 1 (control group), Central region = 2, Western region = 3		

**Table 2:** Descriptive statistics of categorical variables.

Variables	Components	Percentage
TikTok use	Use	13.6%
	Not use	86.4%
Protein intake	Yes	78.9%
	No	21.1%
Gender	Male	48.8%
	Female	51.2%
Marriage	Married	82.9%
	Unmarried	17.1%
Urban	Urban	47.1%
	Rural	52.9%
Geographic region	Eastern region	44.0%
	Central region	29.4%
	Western region	26.6%

**Table 3:** Multiple regression results.

Dependent Variable	Self-Rated Health	
**Independent Variable**	**Coefficient**	**Robust SE**
TikTok use	0.125 **	0.054
**Control Variables**	**Coefficients**	**Robust SE**
Age	−0.012 ***	0.004
Gender	0.215 ***	0.039
Marriage	−0.017	0.054
Subjective social status	0.033	0.021
Subjective economic status	0.180 ***	0.021
BMI	0.009	0.048
BMI squared	−0.000	0.001
Sleep duration a day	0.284 ***	0.069
Sleep duration a day squared	−0.018 ***	0.005
Number of family members	0.019 **	0.009
Ratio of family medical expenditure	−0.424 ***	0.156
Urban	0.095 **	0.040
Geographic region: Central region	0.032	0.045
Geographic region: Western region	−0.149 ***	0.049
Constant	1.468 **	0.687
Observations	4115	
R-squared	0.071	

Note: *** *p* < 0.01, ** *p* < 0.05.

**Table 4:** Stepwise regression results.

Dependent Variables	Exercise Frequency		Self-Rated Health		Protein Intake		Self-Rated Health	
**Independent Variable**	**Coefficient**	**Robust SE**	**Coefficient**	**Robust SE**	**Coefficient**	**Robust SE**	**Coefficient**	**Robust SE**
TikTok use	1.037 ***	0.130	0.116 **	0.055	0.628 ***	0.138	0.115 **	0.054
**Mediating Variables**	**Coefficients**	**Robust SE**	**Coefficients**	**Robust SE**	**Coefficients**	**Robust SE**	**Coefficients**	**Robust SE**
Exercise frequency			0.009	0.007				
Protein intake							0.120 **	0.049
**Control variables**	YES		YES		YES		YES	
Constant	−4.190 ***	1.200	1.506 **	0.685	−2.330 **	1.034	1.418 **	0.693
Observations	4115		4115		4114		4114	
R-squared	0.102		0.071		0.039		0.072	

Note: *** *p* < 0.01, ** *p* < 0.05.

**Table 5:** Generalized structural equation model results.

	Effect Value	Boot S.E.	95% CI Lower Limit	95% CI Upper Limit
Direct effect	0.086	0.044	0.004	0.169
Indirect effect1 (Exercise frequency)	0.006	0.006	−0.006	0.019
Indirect effect2 (Protein intake)	0.058	0.029	0.005	0.125
Total effect	0.150	0.052	0.043	0.251

Note: bootstrap sampling 500 times.

**Table 6:** Robustness test results.

Dependent Variables	Self-Rated Health		ADL	
**Independent Variable**	**Coefficients**	**Robust SE**	**Coefficients**	**Robust SE**
TikTok use	0.090 *	0.054	0.110 **	0.049
Subjective wellbeing	0.097 ***	0.009		
**Control variables**	YES		YES	
Constant	1.350 **	0.655	6.264 ***	1.104
Observations	4115		3191	
R-squared	0.096		0.094	

Note: *** *p* < 0.01, ** *p* < 0.05, * *p* < 0.1.

**Table 7:** Endogenous test results.

Dependent Variables	TikTok Use		Self-Rated Health	
**Independent Variable**	**Coefficients**	**Robust SE**	**Coefficients**	**Robust SE**
TikTok use			1.033 **	0.446
IMR			−0.502 **	0.246
**Control variables**	YES		YES	
Constant	3.053 ***	0.352	0.624	0.624
Observations	4637		4115	
R-squared	0.094		0.072	

Note: *** *p* < 0.01, ** *p* < 0.05.

## Data Availability

The datasets presented in this study are publicly available via the CFPS database, http://www.isss.pku.edu.cn/cfps/ (accessed on 30 June 2023). Further queries can be directed to the authors.
